# Arthro‐Cross Compression Technique for Anterior Cruciate Ligament Avulsion Fracture Fixation

**DOI:** 10.1002/atn2.70311

**Published:** 2026-10-15

**Authors:** Krishan Kumar, Vishesh Verma, Mukesh Garg, Vineet Pruthi, Jaiveer Singh

**Affiliations:** ^1^ ESIC Medical College and Hospital Faridabad India; ^2^ Sarvodaya Healthcare Faridabad India

## Abstract

Isolated anterior cruciate ligament avulsion fractures of the tibial eminence are uncommon in orthopaedic practice. These injuries were traditionally treated with open reduction and internal fixation, but arthrotomy‐related morbidity has led to an increased use of arthroscopic techniques. Consequently, arthroscopic fixation techniques have gained popularity because of reduced surgical morbidity. In this article, we describe a simple arthroscopic procedure that ensures near‐anatomic reduction with even circumferential compression and no fracture fragment lift‐off.

## Graphical abstract

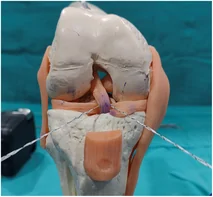

**VIDEO 1:** Narrated video (right knee, patient supine, knee flexed at 90°, standard anterolateral viewing portal unless specified) showing the Arthro‐Cross Compression Technique for arthroscopic anterior cruciate ligament avulsion fracture fixation. Key learning points include debridement of the fracture crater and fragment for clean bed preparation; provisional Kirschner wire reduction; secure Bio‐Fiber tape passage through the anterior cruciate ligament base to avoid comminution; precise dual tibial tunnel drilling (anteromedial and anterolateral exits); shuttling for crossed tape (rotational stability) and noncrossed anterior suture (broad compression); sequential tensioning in 30° flexion over EndoButton; and final construct showing no lift‐off. Video content can be viewed at https://doi.org/10.1002/atn2.70311.

The incidence of isolated anterior cruciate ligament (ACL) avulsion fracture is estimated at 3 per 100,000 annually.^1^ This rate has increased because of rising athletic activities and road traffic accidents.^2^ Fixation can be achieved using various implants, but arthroscopic techniques with screws or sutures are mainstream.^3^

Arthroscopic reduction and internal fixation of ACL avulsions with screws was first described in 1993 by Lubowitz and Grauer,^4^ but disadvantages include fragment comminution, posterior neurovascular injury, and hardware removal needs.^5^ Nonabsorbable sutures are optimal.^6^ Biomechanical studies on suture versus screw fixation remain inconclusive: some favor screws for rigidity under cyclic loading, others show no differences, and some indicate that sutures provide greater strength.^7^

Most arthroscopic techniques involve passing a tying suture at the ACL base and pulling it through single or double interosseous tunnels. However, for large or comminuted fragments, these constructs may result in nonanatomic reduction, uneven compression, and anterior lift‐off.

The Arthro‐Cross Compression Technique (ACT) is an arthroscopic method for tibial‐sided ACL avulsion fractures. It uses a crossed No. 5 Bio‐Fiber tape passed through the ACL substance for rotational stability, combined with a noncrossed Bio‐Fiber suture for broad anterior compression, secured extracortically with an EndoButton. ACT provides robust fixation, minimizes hardware complications, and facilitates early mobilization. It adapts to large and comminuted fragments, ensuring near‐anatomic reduction with circumferential compression and no anterior lift‐off.

## SURGICAL TECHNIQUE

### Indications

The indications for this technique include tibial eminence fractures classified as Meyers‐McKeever type II to IV, large single‐fragment avulsions, and comminuted avulsion patterns in which suture‐based fixation is more suitable than screw fixation. It is also indicated in acute injuries where the ACL tissue is preserved and can reliably hold a tape or suture construct.

### Patient Positioning and Arthroscopic Setup

The patient is positioned supine with the knee hanging off the side of the table flexed at 90° (or secured in a leg holder, if available) to optimize arthroscopic access. Standard anterolateral and anteromedial (AM) portals are established for visualization and instrumentation. An accessory AM portal is created for suture retrieval. A cannula can be placed (optional) to streamline suture management.

### Surgical Steps

#### Debridement and Provisional Reduction

The fracture crater and avulsion fragment are debrided using an arthroscopic shaver (4.5‐mm full‐radius shaver, Smith & Nephew, Andover, MA) to ensure a clean reduction bed. The transverse (intermeniscal) ligament is identified and preserved whenever possible; if it limits visualization or reduction of the fragment, it is gently retracted anteriorly with a probe rather than resected. Partial release is performed only when an entrapped intermeniscal ligament prevents anatomic reduction. The fragment is anatomically reduced using an arthroscopic probe and temporarily stabilized with a 2‐mm Kirschner wire (K‐wire; Sironix Medtech, Bengaluru, India) inserted percutaneously. The entire technique is shown in Video 1.

#### Bio‐Fiber Tape Passage Through ACL

A No. 5 Bio‐Fiber tape (Sironix Medtech), composed of braided ultrahigh molecular weight polyethylene, is passed around the base of the ACL using a 45° curved suture lasso (Sironix Medtech), ensuring secure anchorage within the ligament substance (Figure 1).

**FIGURE 1:**
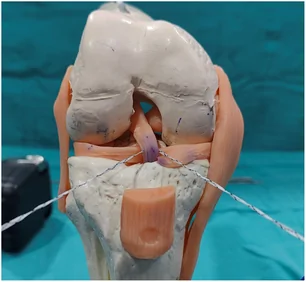
Sawbones model of the right knee (simulating supine position, knee flexed at 90°) showing the passage of No. 5 Bio‐Fiber tape through the base of the ACL stump using a suture lasso device. This step illustrates secure anchorage within the ligament substance, avoiding the risk of further fragment comminution in a synthetic tibial eminence avulsion setup. (ACL, anterior cruciate ligament.)

#### Tibial Tunnel Drilling

Two 2.4‐mm tibial tunnels are drilled using Beath pins (Sironix Medtech), with an ACL tibial guide (Sironix Medtech) set at a 60° angle. Entry points are positioned 1 cm apart on the AM tibia, with intra‐articular exits at the AM and anterolateral aspects of the fracture crater (Figure 2). For large single fragments, tunnels may be drilled directly through the fragment to enhance fixation stability.

**FIGURE 2:**
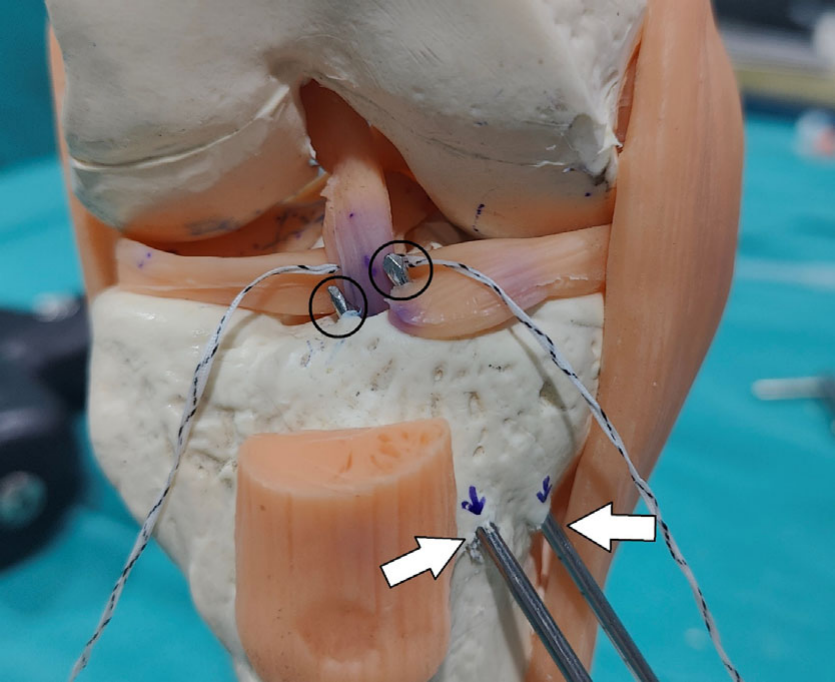
Sawbones model of the right knee (simulating supine position, knee flexed at 90°) showing the Beath pin placement over the medial proximal tibia, ensuring a cortical bridge. The intra‐articular exit is positioned over the anteromedial and anterolateral aspect of the fracture crater in the synthetic model.

#### Suture and Tape Shuttling

An 18G spinal needle loaded with a No. 1 Prolene suture (Ethicon, Johnson & Johnson, Somerville, NJ) is passed through the AM tunnel after removing the Beath pin. The Prolene suture is retrieved via the accessory AM portal (Figure 3).

**FIGURE 3:**
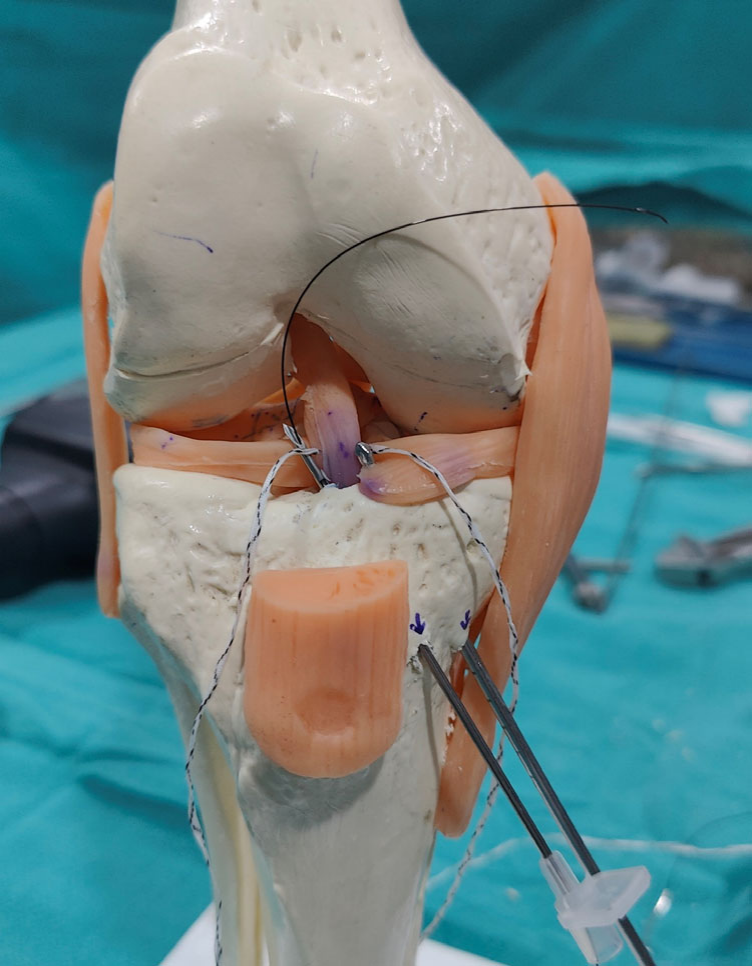
Sawbones model of the right knee (simulating supine position, knee flexed at 90°) illustrating a spinal needle replacing the Beath pin for shuttling the Bio‐Fiber tape (in crossed configuration) and suture (in noncrossed configuration). The needle is advanced through the anteromedial tunnel, with Prolene suture retrieval via the accessory anteromedial portal.

The lateral end of the Bio‐Fiber tape (from the lateral side of the ACL) is retrieved through the same portal, ensuring a crossed configuration (the lateral Bio‐Fiber tape end exits the AM tunnel). One end of a 5‐mm Bio‐Fiber suture (Sironix Medtech) is tied to the Prolene suture at a distance from the Bio‐Fiber tape.

The Prolene suture is pulled through the AM tunnel, shuttling the Bio‐Fiber tape and Bio‐Fiber suture. This process is repeated for the anterolateral tunnel, retrieving the medial Bio‐Fiber tape end and the opposite Bio‐Fiber suture end. This creates a noncrossed Bio‐Fiber suture and a crossed Bio‐Fiber tape configuration (Figure 4).

**FIGURE 4:**
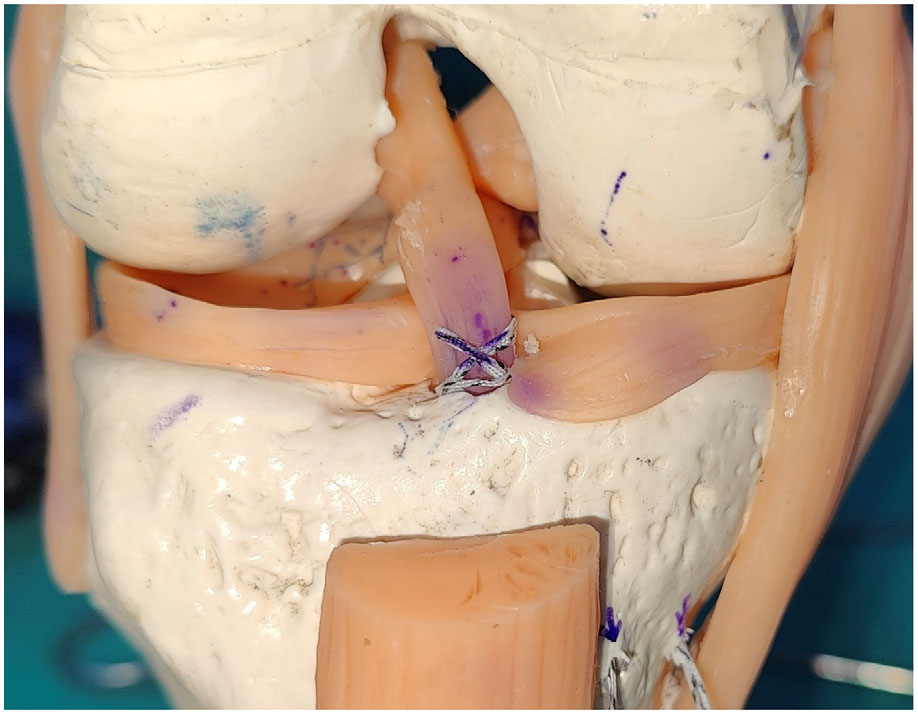
Sawbones model illustration of the configuration after Bio‐Fiber suture and tape passage. The Bio‐Fiber tape is shown in a crossed pattern, passing through the ACL base and exiting opposite tibial tunnels to enhance rotational stability. The Bio‐Fiber suture is noncrossed and positioned anteriorly for broad compression over the avulsed fragment. (ACL, anterior cruciate ligament.)

#### Fixation and Tensioning

Both Bio‐Fiber suture and Bio‐Fiber tape are loaded onto an OPENBUTTON EndoButton (Sironix Medtech). With the knee in 30° flexion, the Bio‐Fiber tape is tensioned first to stabilize the ACL, and knots are tied after removing the K‐wire. The Bio‐Fiber suture is then tensioned, creating a broad anterior compression zone over the fragment without passing through the ACL (Figure 5). Although the construct can also be performed with 2 Bio‐Fiber tapes, the combination of 1 tape (for the crossed intraligamentous limb that requires the greatest mechanical strength and broad contact area at the ACL base) and 1 Bio‐Fiber suture (for the anterior compression limb) was chosen primarily for cost‐effectiveness because a No. 5 Bio‐Fiber tape is substantially more expensive than a Bio‐Fiber suture. In our experience, the lower‐profile suture also provides sufficient anterior compression once the tape limb has already been tensioned without compromising the overall construct stability. Surgical pearls and pitfalls are summarized in Table 1.

**FIGURE 5:**
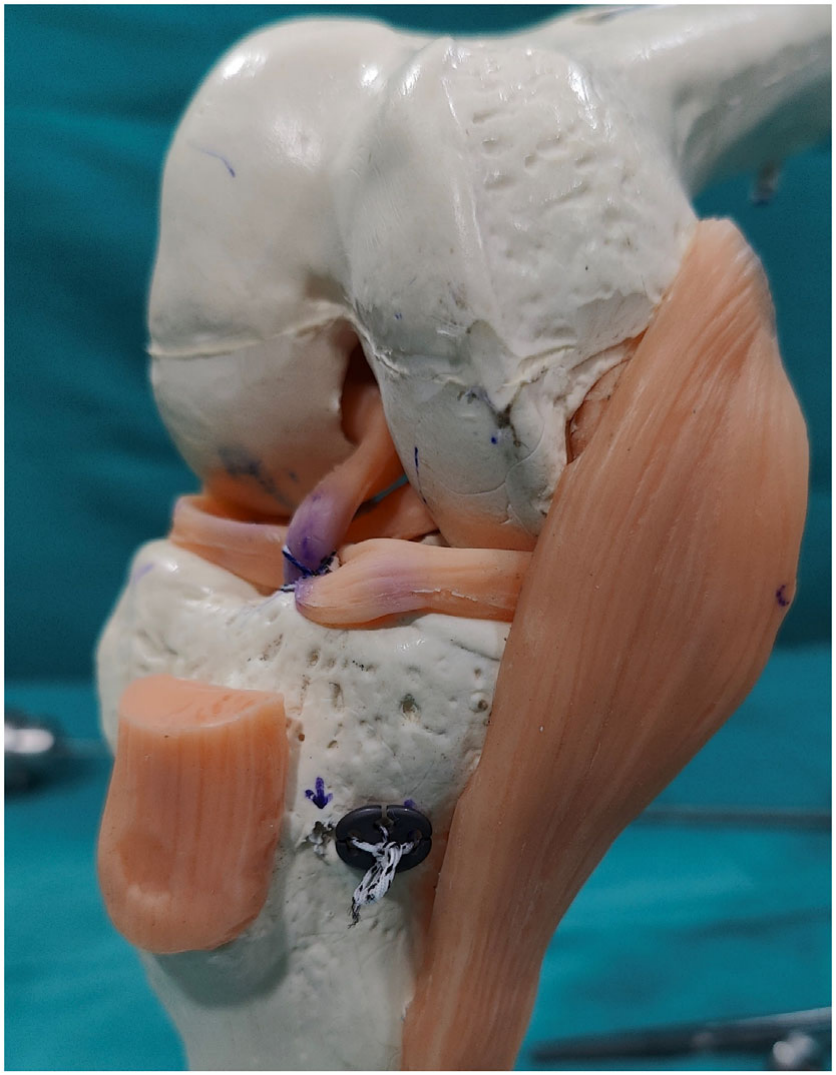
Sawbones model illustration of the final construct after tying over the cortical button (EndoButton). The crossed Bio‐Fiber tape provides ACL stabilization, whereas the noncrossed suture delivers anterior compression without traversing the ligament, secured extracortically in the synthetic tibia. (ACL, anterior cruciate ligament.)

**TABLE 1:** Surgical Pearls and Pitfalls

**Pearls**	**Pitfalls**
• Passing the tape through the ACL stump along with the fragment prevents further comminution and stabilizes small fragments • Precise tunnel placement using the ACL jig prevents convergence or cortical breach, ensuring optimal suture trajectory • Tension the Bio‐Fiber tape before the Bio‐Fiber suture to prioritize ligament stability, followed by fragment compression • Tying the suture and tape over a cortical EndoButton avoids the direct cutting impact over the bony bridge	• Overtightening of Bio‐Fiber suture may cause fragment tilt; confirm reduction arthroscopically before final knotting • In comminuted fractures, ensure tape passage captures major fragments to prevent displacement

ACL, anterior cruciate ligament.

#### Final Construct

The Bio‐Fiber tape forms a crossed configuration, passing through the ACL base and exiting through opposite tibial tunnels, enhancing rotational stability. The Bio‐Fiber suture, positioned anteriorly in a noncrossed configuration, provides a wide compression construct, reducing the fragment and minimizing anterior tilt, secured extracortically by the OPENBUTTON EndoButton (Figures 6 and 7 show pre‐ and postoperative radiographs of a patient managed with this technique).

**FIGURE 6:**
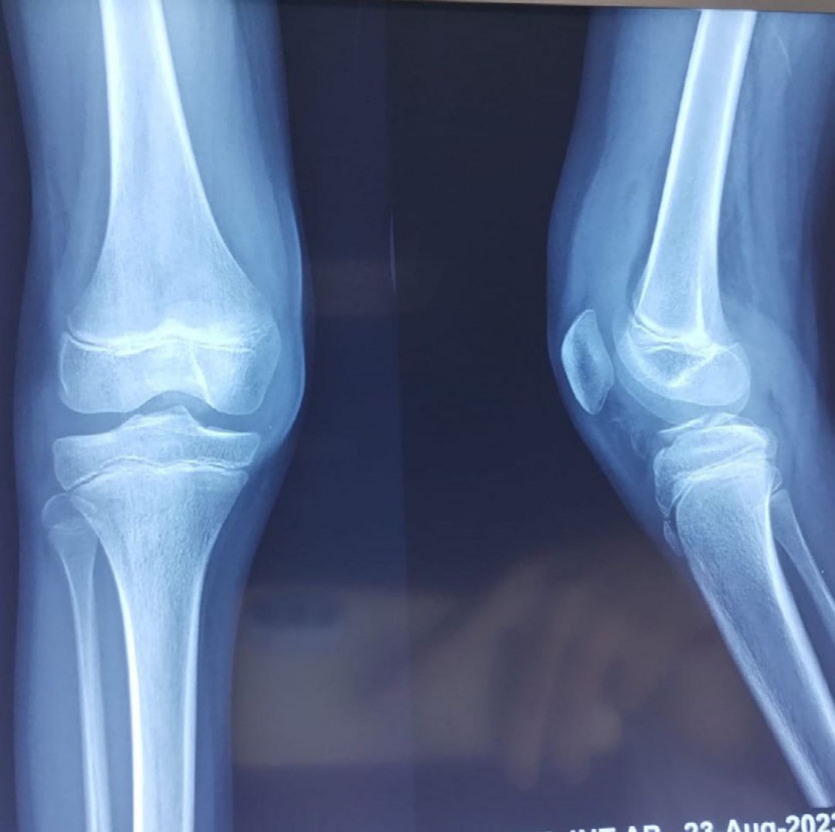
Preoperative anteroposterior and lateral radiographs of the right knee showing the ACL avulsion fracture with displacement of the tibial eminence fragment. (ACL, anterior cruciate ligament.)

**FIGURE 7:**
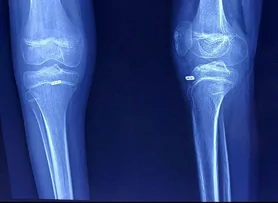
Postoperative anteroposterior and lateral radiographs of the right knee showing near‐anatomic reduction of the ACL avulsion fracture without lift‐off, achieved using the Arthro‐Cross Compression Technique. (ACL, anterior cruciate ligament.)

### Postoperative Protocol

The knee is immobilized in a hinged brace for 2 weeks, allowing 0° to 30° flexion to protect the repair. Partial weight‐bearing begins at 2 weeks, progressing to full weight‐bearing by 4 to 6 weeks, guided by clinical and radiographic assessment. Physiotherapy, including range‐of‐motion and strengthening exercises, commences at 4 weeks, with return to activity targeted at 3 to 6 months based on healing progress.

## DISCUSSION

The optimal fixation strategy for tibial eminence ACL avulsion fractures remains debated because of variable fragment morphology, chronicity, soft‐tissue conditions, and patient factors. Before arthroscopic techniques, these fractures were treated with open reduction and internal fixation using screws, K‐wires, or staples, risking joint exposure and infection when the skin condition was poor. McLennan explored arthroscopic‐assisted fixation to minimize soft‐tissue trauma.^8^

Interfragmentary screws, staples, and K‐wires offer rigidity but risk loosening, backout, and impingement in the metaphyseal tibia, especially with the anterior ACL footprint.^9^ Comminuted fragments pose challenges for bony purchase, and hardware removal is often needed.

Sutures or anchors avoid these issues. Anchor backout remains a concern in cancellous proximal tibia with post‐trauma edema, making nonabsorbable sutures preferable.^6^ Suture fixation typically involves passing through the ACL or fragment and tying over a bone bridge or cortical button.^10^

In small or comminuted fragments, secure purchase from the fragment alone is unreliable; incorporating the ACL stump improves stability. In large single fragments, anterior lift‐off may occur with 1 or 2 tunnels, and although 4‐tunnel constructs reduce this, they are technically demanding.^11^

ACT uses 2 tibial tunnels with independent tensioning of a crossed tape and a noncrossed anterior suture, providing modular fixation, improved rotational control, and broad anterior compression. This construct supports early rehabilitation because of its stability, although biomechanical studies are needed to validate its superiority.

Several arthroscopic suture‐based constructs for tibial eminence avulsion fixation have been described that share conceptual similarities with ACT. DeFroda et al. reported an anchorless 2‐tunnel pull‐through suture technique using double Hewson suture passers, with sutures tied over a bone bridge.^12^ More recently, Morag et al. described a modified triangular pull‐through suture‐based fixation for type II and III avulsions, showing favorable International Knee Documentation Committee and Tegner scores at a minimum 2‐year follow‐up.^13^ ACT differs from these constructs in 3 respects: it uses a crossed ultrahigh molecular weight polyethylene tape rather than round sutures alone, providing a broader contact area at the ACL footprint and reducing the risk of cheese‐wire effect through the ligament; it adds an independent noncrossed anterior suture limb that delivers plate‐like compression over the fragment without traversing the ACL; and it secures both limbs extracortically over a single cortical button, avoiding the need for a bone‐bridge tie‐over and simplifying tensioning. The complete advantages and disadvantages of ACT relative to the existing techniques are summarized in Table 2.

**TABLE 2:** Advantages and Disadvantages

**Advantages**	**Disadvantages**
• Simple technique • Cost‐effective • The crossed Bio‐Fiber configuration enhances rotational stability, addressing shear forces during knee motion • The noncrossed Bio‐Fiber suture provides broad compression, mimicking a plate‐like effect to promote fragment healing • Minimal hardware (only 1 cortical EndoButton) reduces complications associated with screws or anchors • The technique is versatile, and it accommodates both large single fragment and comminuted fractures through adaptable tunnel placement • Robust fixation supports early mobilization and may improve functional outcomes	• Needs further biomechanical studies to validate superiority • Demands a certain level of arthroscopic learning curve and understanding

## DECLARATION OF GENERATIVE AI AND AI‐ASSISTED TECHNOLOGIES IN THE WRITING PROCESS

During the preparation of this work, the authors used ChatGPT (GPT‐5.2) and Anthropic Claude Opus 4.7 to assist with language refinement, grammar correction, and formatting according to journal guidelines. After using this tool, the authors reviewed and edited the content as necessary and take full responsibility for the final manuscript.

## DISCLOSURES

The authors (K.K., V.V., M.G., V.P., J.S.) declare that they have no known competing financial interests or personal relationships that could have appeared to influence the work reported in this article.
